# Unravelling the impact of ethnicity on health in Europe: the HELIUS study

**DOI:** 10.1186/1471-2458-13-402

**Published:** 2013-04-27

**Authors:** Karien Stronks, Marieke B Snijder, Ron JG Peters, Maria Prins, Aart H Schene, Aeilko H Zwinderman

**Affiliations:** 1 Department of Public Health, Academic Medical Center, University of Amsterdam, Amsterdam, The Netherlands; 2 Department of Cardiology, Academic Medical Center, University of Amsterdam, Amsterdam, The Netherlands; 3 Public Health Service of Amsterdam, Amsterdam, The Netherlands; 4 Department of Internal Medicine, Center for Infection and Immunity Amsterdam (CINIMA), Academic Medical Center, University of Amsterdam, Amsterdam, The Netherlands; 5 Department of Psychiatry, Academic Medical Center, University of Amsterdam, Amsterdam, The Netherlands; 6 Department of Clinical Epidemiology and Biostatistics, Academic Medical Center, University of Amsterdam, Amsterdam, The Netherlands

**Keywords:** Ethnicity, Prospective cohort study, Design, Cardiovascular health, Mental health, Infectious diseases

## Abstract

**Background:**

Populations in Europe are becoming increasingly ethnically diverse, and health risks differ between ethnic groups. The aim of the HELIUS (HEalthy LIfe in an Urban Setting) study is to unravel the mechanisms underlying the impact of ethnicity on communicable and non-communicable diseases.

**Methods/design:**

HELIUS is a large-scale prospective cohort study being carried out in Amsterdam, the Netherlands. The sample is made up of Amsterdam residents of Surinamese (with Afro-Caribbean Surinamese and South Asian-Surinamese as the main ethnic groups), Turkish, Moroccan, Ghanaian, and ethnic Dutch origin. HELIUS focuses on three disease categories: cardiovascular disease (including diabetes), mental health (depressive disorders and substance use disorders), and infectious diseases. The explanatory mechanisms being studied include genetic profile, culture, migration history, ethnic identity, socio-economic factors and discrimination. These might affect disease risks through specific risk factors including health-related behaviour and living and working conditions. Every five years, participants complete a standardized questionnaire and undergo a medical examination. Biological samples are obtained for diagnostic tests and storage. Participants’ data are linked to morbidity and mortality registries. The aim is to recruit a minimum of 5,000 respondents per ethnic group, to a total of 30,000 participants.

**Discussion:**

This paper describes the rationale, conceptual framework, and design and methods of the HELIUS study. HELIUS will contribute to an understanding of inequalities in health between ethnic groups and the mechanisms that link ethnicity to health in Europe.

## Background

Prospective population-based cohort studies have significantly increased our understanding of risk factors for the major causes of the global burden of disease. This knowledge has provided a firm basis for effective preventive and treatment strategies. For example, the identification of the main risk factors for cardiovascular disease (such as hypertension and smoking) in the Framingham Heart Study has formed the basis for a dramatic reduction in cardiovascular morbidity and mortality since the 1970s [1].

Most large population-based cohort studies have excluded ethnic minority populations [2]. The main arguments in favour of ethnic homogeneity relate to the internal validity and statistical power of the study. The more homogenous the study population, the more likely it will be that the observed associations have not been biased due to confounding introduced by a multi-ethnic population. However, the more homogeneous the study population, the less closely it will resemble the heterogeneity of the ‘real-world’ population. These real-world populations in Europe are increasingly ethnically diverse. In the Netherlands, for example, approximately 10% of the population is of non-Western origin. This will increase to 20% by 2060 [3].

Often, the disease risk profile of ethnic minority populations differs from that of the majority population – sometimes in favour, but mostly to the detriment of minority populations. Well-documented examples are a higher prevalence of coronary heart disease among people originating from the South Asian subcontinent [4], a higher prevalence of depression among labour migrants from Turkey [5], a higher prevalence of stroke among people originating from Africa [6], and higher death risks from almost all infectious diseases among ethnic minorities [7]. This pattern of ethnic health inequalities applies to migrants as well as to their offspring, with inequalities sometimes increasing and sometimes decreasing across generations. In addition, there is evidence that risk factor-outcome relationships differ across ethnic groups. For example, in case of the relationship between overweight and the risk of cardiovascular disease, optimal body mass index seems to be lower for South Asian populations [8]. Associations between specific genetic variants and complex chronic diseases also vary between ethnic groups: for example, 5 out of 19 common single-nucleotide changes found to be strongly related to type 2 diabetes in European populations show different associations in other ethnic groups [9].

Because of these epidemiological differences, the findings of published cohort studies cannot automatically be generalized to the European population as a whole. In addition, due to a lack of studies that include a substantial number of respondents from ethnic minority groups, current cohort studies do not allow for an exploration of inequalities in health between ethnic groups. Therefore, there is an urgent need for population-based cohort studies that include substantial numbers of different ethnic groups, including a comparison group from the host population [2,10]. These studies should acknowledge the dynamic character of disease patterns and thus include several generations.

HELIUS (acronym for HEalthy LIfe in an Urban Setting) is a prospective cohort study that aims to fill this gap in our knowledge (http://www.heliusstudy.nl/en/home). The aim is to unravel the causes of the unequal burden of disease across ethnic groups, and ultimately enable the improvement of health care and prevention strategies. The study is being carried out in Amsterdam, the Netherlands, and is an initiative of the Academic Medical Center (AMC) and the Public Health Service of Amsterdam (GGD Amsterdam). This paper outlines the conceptual framework underlying the HELIUS study as well as the design and methods used.

### Design: conceptual framework

In this section, we will first define the concept of ethnicity. We will then describe the conceptual framework underlying HELIUS, which specifies how ethnicity is linked to health.

Until the second half of the twentieth century, race – as defined by physical appearance characteristics such as skin colour, eye shape, and facial structure – was considered a useful way of classifying people into ethnic groups. Current knowledge challenges this view, as there are no validated biological criteria on a phenotype level to determine an individual’s race. The concept of ethnicity has therefore overtaken race in medical sciences [11]. In Europe, there is a lack of an agreed upon definition of ethnicity. Current definitions all have in common that they define ethnic groups as a social construct. More specifically, an ethnic group is defined as a group that has a shared history, ancestry, and identity, and that shares characteristics such as a geographical affiliation, culture and traditions, language, and religious tradition [12].

The way this complex concept of ethnicity is translated into statistical indicators differs between European countries. Frequently used indicators include country of birth, self-identified ethnicity, and nationality [12]. In the Netherlands, country of birth has become widely accepted as a basis for identifying ethnic groups. This classification, which is the result of a long and intensive discussion, defines ethnic groups according to an individual’s country of birth as well as that of his or her parents. This is the definition used in HELIUS. Specifically, a person is defined as of non-Dutch ethnic origin if she or he fulfils one of two criteria: he or she was born outside the Netherlands and has at least one parent who was born outside the Netherlands (first generation); or he or she was born in the Netherlands but both parents were born outside the Netherlands (second generation) [13].

The HELIUS study is based on the assumption that an individual’s ethnic background influences health on two levels. First, ethnicity is associated with an uneven distribution of specific risk factors, also called proximal factors as they are considered to be proximate to the onset of pathogenic processes. These include physical (e.g. working conditions), behavioural (e.g. smoking), psychosocial (e.g. stress), and biological (e.g. hypertension) exposures that trigger pathogenic processes, and can therefore be perceived as a direct cause of a disease [14]. If the outcome measure is prognosis rather than the incidence of a certain disease, the proximal risk factors also include health care [15]. Which of these risk factors should be considered relevant depends on the specific outcome measure under study. For example, ethnic inequalities in the incidence of type 2 diabetes might be due to an uneven distribution of behavioural risk factors such as dietary habits or lack of exercise, whereas ethnic inequalities in depression might arise from an uneven distribution of psychosocial factors such as stressors across ethnic groups.

Second, it is important to examine the causal pathways that link the concept of ethnicity to the risk factors that are proximate to diseases. It is no coincidence that these risk factors are unevenly distributed across ethnic groups: this distribution is rooted in these ethnic groups’ characteristics, such as their genetic profile, cultural orientation, and the social conditions they are exposed to as a result of migration [13,16]. As such, ethnicity can be considered an ‘umbrella construct’ that comprises many different aspects [17]. A distinction can be made between two dimensions within these aspects: an attributional dimension, which describes the unique characteristics of a certain ethnic group, such as genetic profile or cultural orientation, and a relational dimension, which captures the characteristics of the relationship between a certain ethnic group and the society that group lives in, including discrimination and socio-economic position [17]. Although essentially attributional or relational in character, most of these characteristics can be used either attributionally or relationally. For example, ethnic identity is a characteristic of a certain group, but at the same time is formed by the relationships this group has with other groups in the host country. These characteristics thus explain why a certain proximal risk factor is unevenly distributed across ethnic groups. If, for example, a certain ethnic minority group has an increased risk of smoking, this might be due to the fact that the group is exposed to discrimination in the host country (relational dimension), or to specific sociocultural values characteristic for that group (attributional dimension). By placing the distribution of proximal factors in these causal pathways, scientific research will yield statements on the explanation of ethnic inequalities in health that are generalizable to other ethnic groups with similar characteristics.

**Figure 1:**
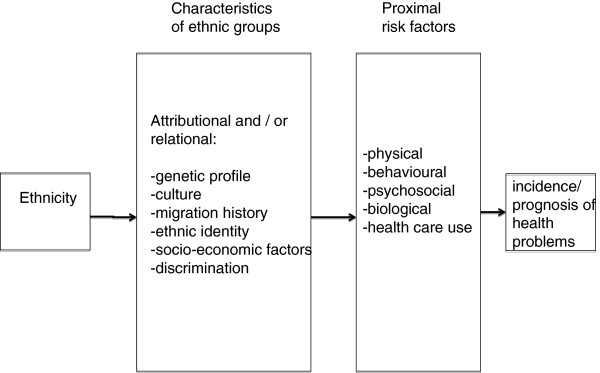
Conceptual framework integrating possible explanations for the relationship between ethnicity and health.

The literature on ethnicity and health indicates at least the following pathways that link ethnicity to health through proximal risk factors: genetic profile, culture, migration history, ethnic identity, socio-economic position, and discrimination [17-20]. They influence the incidence and prognosis of diseases through specific proximal risk factors. The idea is that by testing specific hypotheses as to the explanatory mechanisms underlying the association between ethnicity and health outcomes, the concept of ethnicity might come to be replaced by the explanatory characteristics associated with this [21]. This is visualized in Figure 1. Each of the causal pathways will be discussed below.

### Genetic profile

Ethnic groups vary in the frequency of certain genetic diseases. Examples include haemoglobinopathies (thalassaemias and sickle cell disease), which are more common in populations from most Arab countries [22]. These conditions are relatively rare, however. With respect to common, complex diseases such as type 2 diabetes, there is evidence that the contribution of single genes to the ethnic variation in risks is limited [23]. This does not exclude the possibility that genetics plays a role in ethnic variation in specific health problems, such as the susceptibility to viral infections [24]. In addition, interactions between genes and environment (epigenetics) may contribute to excess risk of, for example, high blood pressure or diabetes mellitus [25]. ‘Thrifty genotypes’ may be the underlying mechanism of the gene and environmental interactions that contribute to disease susceptibility [26]. Further genetic studies are warranted to support this view.

### Culture

Culture can be seen as a ‘lens’ through which an individual perceives, interprets, and copes with the world or environment in which he or she lives. It includes norms, values, and beliefs that are socially transmitted. These aspects might affect health through health-related behaviour and health care use in particular [27,28].

The role of cultural factors in the association between ethnicity and health is seldom studied directly. Instead, ethnicity is often used synonymously with culture, and the association between ethnicity and health that cannot be explained by such things as socio-economic status is then assumed to reflect the influence of culture [27]. This approach may be too simple, however, as the association between ethnicity and health is potentially mediated by many factors, as indicated in Figure 1.

Because of the lack of empirical studies that actually measure cultural factors and specify the pathways that link culture to health, no conclusions can be drawn on the importance of culture as an explanation for ethnic inequalities in health [29]. In addition, it is important to realize that the importance of culture to health might differ between outcome measures due to the fact that culture might serve both as a health protective factor (e.g. religious values that reduce alcohol use) and a factor that increases the risk of health problems (e.g. social norms that increase the risk of smoking). When considering the role of culture, its dynamic character should also be taken into account. The cultures of ethnic minorities are constantly changing during the process of contact with the host population, something that is usually referred to as acculturation. However, the measurement of this concept in current studies is far from perfect. Criticisms include the lack of a theoretical framework, the use of simple proxy measures such as length of stay, the use of unidirectional and incomplete instruments, and the reduction of culture to a characteristic of the individual, independent of his or her social and material context [30,31].

Future studies of the role of cultural factors thus requires that these factors be investigated directly, using measures based on a theoretical model that specifies the causal pathway that links culture to a specific health outcome. In addition, it should be acknowledged that the culture of an ethnic minority population has a dynamic character, and is influenced by the host population as well as by contacts with the country of origin. Finally, the role of a cultural explanation should be assessed in comparison to other potentially relevant explanatory mechanisms.

### Migration history

The Dutch classification of ethnicity in terms of country of birth can be viewed at least in part as an index of migration, given the distinction that can be made between first generation (immigrants) and second generation (offspring of immigrants). Other dimensions of migration history are not captured by the country of birth classification. Additional indicators might provide further insight into the differences in migration history of people who share the same country of birth. These include:

•The place (and/or type) of residence or region where a person grew up. For example, an increase in the risk of cardiovascular disease has been observed in people in developing countries who migrated from rural to urban areas, probably as a result of changes in environment and lifestyle [32].

•The time of residence in the host country. This might be a useful indicator for describing such things as similarities between first-generation migrants who migrated at a very young age and second-generation migrants who were born in that country.

•The dynamics of the relationships with family in the country of origin, which might also act as a stressor [33].

### Ethnic identity

Probably the most well-known definition of ethnicity is that of Weber, who states that ‘We shall call “ethnic groups” those human groups that entertain a subjective belief in their common descent because of similarities of physical type or of customs or both, or because of memories of colonization and migration’ [34]. Inherent in this definition of ethnicity is that individuals establish their own ethnic *identity*. Ethnic identity indicates the individual’s feelings or emotional attachment towards a specific ethnic group [35]. It can therefore be considered the ‘psychological label’ that an individual attaches to him- or herself. This is a fluid label, in the sense that it might vary over time and is shaped by the social context people live in, including their relationship with other ethnic groups in society. In addition, it might vary across domains of life [17].

Identity is clearly a dimension of ethnicity in that it influences the occurrence of health problems, operating through a psychosocial or behavioural mechanism, social participation, or buffering the effects of discrimination [36,37]. We are not aware of studies that estimate the relative importance of this component in relation to ethnic inequalities in health. Further empirical research is necessary, based on specified hypotheses that indicate the way ethnic identity influences health problems.

### Socio-economic factors

Ethnic minority groups have a lower average socio-economic status than the host population. Given the association between socio-economic status and health, it is likely that ethnic inequalities in health are socio-economic in nature, at least in part [18,19]. The health effect of socio-economic status is established through a range of specific risk factors, including physical living and working conditions and health-related behaviour.

Differences in socio-economic status are frequently considered a potential source of *bias* in studies on ethnic inequalities in health, as a confounder of the real effect of ethnicity [38]. To assess the ‘real’ effect, data are usually presented after adjustment for differences in socio-economic status. The conceptual model as visualized in Figure 1 indicates a different perspective: it postulates that ethnic groups differ in a broad range of characteristics, including genetic profile, culture, and socio-economic status. It is through all these characteristics that the influence of ethnic background on health is expressed. This implies that these characteristics should all be handled as explanatory variables, including socio-economic factors [38].

The results of previous studies on the role of socio-economic indicators indicate that ethnic differences are greatly reduced – and sometimes even disappear – when socio-economic factors are taken into account. However, many questions remain. These include the variation between ethnic groups in the way socio-economic characteristics and health are related [39], and the validity of the measurement of socio-economic status among different ethnic groups [40].

### Discrimination

Discrimination in the context of ethnicity can be described as unequal treatment of individuals from different ethnic backgrounds. Institutional discrimination involves discriminatory policies or practices carried out by institutions, resulting in such things as an adverse socio-economic position or inadequate health care. Interpersonal discrimination refers to directly perceived discriminatory interactions between individuals, including insults or unfair treatment at work or in public places.

As with culture, discrimination has been inferred as a possible explanation after socio-economic factors have been taken into account. Studies that actually assess discrimination indicate a relationship between experiences of interpersonal discrimination and perceived general health, mental health problems, and raised blood pressure [41,42]. Many questions remain, however, including the extent to which institutional discrimination underlies socio-economic disadvantage or an impaired quantity and/or quality of health care, the specific mechanisms through which discrimination is linked to health, and the role of contextual factors such as ethnic identity. Further empirical studies are needed to explore these issues.

## Methods/design

### Study objectives

The objective of the HELIUS study is to assess ethnic inequalities in the incidence and prognosis of major diseases, and to analyse the causes of these inequalities. The ultimate aim is to contribute to the evidence base for the improvement of health care and prevention strategies in different ethnic groups. HELIUS focuses on three of the major causes of the global burden of disease: cardiovascular disease (including diabetes), mental health (in particular, depressive disorders and substance use disorders), and infectious diseases. With regard to the explanations for ethnic inequalities in health, HELIUS focuses on proximal risk factors (health-related behaviour, working and housing conditions, health care, etc.) as well as on the underlying mechanisms as specified in the conceptual model presented in the previous section (socio-economic factors, genetic profile, culture, etc.).

### Design and study participants

The HELIUS study was designed as a prospective cohort study, and is being carried out in Amsterdam, the Netherlands. It includes people with an ethnic Dutch background along with individuals with Afro-Caribbean Surinamese, South Asian-Surinamese, Turkish, Moroccan, and Ghanaian backgrounds. The ethnic minority groups included in the HELIUS study are the largest ethnic minority groups in Amsterdam. Many ethnic minorities in the Netherlands live in the large cities: 35% of the present inhabitants of Amsterdam are of non-Western origin, with the largest subgroups being made up of people of Moroccan, Surinamese, and Turkish origin [3]. More information on their migration backgrounds can be found in Table 1.

**Table 1:** Information on the migration history of the ethnic minority groups included in HELIUS

**Ethnic group**	**Migration history**
Surinamese	The Surinamese migrated to the Netherlands from Suriname, a former Dutch colony in South America. Surinamese with an Afro-Caribbean background (‘Creole’) are mainly the descendants of West Africans, and those with a South Asian background (‘Hindustani’) have their roots in North India. Both groups migrated to Suriname in the nineteenth century. Their migration from Suriname to the Netherlands was mainly due to the unstable political situation in Suriname in 1975 and 1980. Ethnic minority groups with comparable South Asian and African-Caribbean backgrounds can also be found in other European countries, including the United Kingdom (UK).
Turks and Morrocans	Turks and Moroccans form important migrant groups, not only in the Netherlands but also in other West European countries (Belgium, France, Spain, Italy, and Germany). Migration from Turkey and Morocco was encouraged in the 1960s and early 1970s to fill labour shortages in unskilled occupations. The initial period of labour migration was followed by a second period (1970–1980) in which many guest workers brought their spouses and children to the Netherlands. Since then, many young Turkish and Moroccan people have chosen partners from their region of origin.
Ghanaian	The migration of Ghanaians to the Netherlands occurred in two phases. The first phase (between 1974 and 1983) was due to economic reasons. The second phase (in the early 1990s) was linked to drought, political instability, and the expulsion of Ghanaians from Nigeria. Ghanaians are also an important migrant group in the UK and Germany.

HELIUS is designed to be a multigenerational study. Subjects in the age range of 18 to 70 years are randomly sampled, stratified by ethnic origin, through the municipality registry of Amsterdam (MRA). This registry contains data on the country of birth of residents and their parents, which are needed to determine ethnicity (see definition of ethnicity above). We intend to include a maximum of three relatives per index participant (i.e. a participant included from the MRA sample), which enables us to study family relations as well as different migration generations (based on place of birth). If the parents of an index participant live in Amsterdam, both parents as well as a sibling of the index participant are invited to participate. If an index participant has no parents living in Amsterdam but has one or more children of at least 18 years of age living in Amsterdam, a maximum of two children are invited, as well as the index participant’s partner.

The goal of HELIUS is to include a minimum of 5,000 people per ethnic group. Baseline data collection of HELIUS started in January 2011, and will take approximately five years. Participants will be re-evaluated every five years.

### Ethical issues

The HELIUS study has been approved by the Institutional Review Board of the AMC at the University of Amsterdam. During the informed consent procedure, we ask the participant’s permission 1) to report personal results relevant to the participant’s health to his or her general practitioner (GP); 2) to store his or her biological samples in the HELIUS biobank for future research relating to the three disease groups; 3) to link registries containing data relating to the participant’s health (hospital admissions, pharmacy data, vaccination programmes); 4) to request the official cause of death from Statistics Netherlands (CBS) in the event that the participant dies during the course of the HELIUS study; and 5) to approach him or her for future additional studies and for this to request the municipal registry to update address information.

### Data collection

Data are collected through a questionnaire/interview (I) and a physical examination (II). Biological samples are obtained during study visits (III).

(I) Questionnaire/interview

Individuals in the selected sample receive a written invitation along with written information regarding the study and a response card. If they agree to participate, they can choose to fill in the questionnaire on paper or online. Participants unable to fill in a questionnaire are offered assistance from a trained (ethnically matched) interviewer. The questionnaire addresses the presence (or symptoms) of cardiovascular disease, mental health disorders, and infectious diseases as well as determinants or risk factors of these diseases and disorders (Table 2).

(II) Physical examination

The physical examination takes place at a local research site, after the questionnaire has been completed at home or the interview has taken place. All participants receive a summary of their own main results (blood pressure, lipid profile, glucose and HbA1c levels, renal function, overweight status, and electrocardiogram (ECG) results), accompanied by an explanation and the recommendation to contact their GP if the results are abnormal. The GP receives a copy of these results. If a person does not give permission to forward a copy of the results to the GP, she or he is not allowed to participate in HELIUS.

Table 3 gives an overview of the measurements and laboratory analyses. The collection of biological samples is described in more detail below.

(III) Biological sample collection and testing and storage procedures

**Table 2:** Variables measured in the HELIUS questionnaire

**Theme**	**Explanatory factors**	**Outcomes**
**General**	Demographic factors:	General health, chronic conditions, quality of life (SF-12), functional limitations
	Sex, age, marital status, household composition	
	Ethnicity:	
	Country of birth of respondent and his/her (grand)parents	
	Explanatory mechanisms that link ethnicity to health:	
	Migration history, educational level and occupational status, religion, cultural distance (acculturation), ethnic identity, perceived discrimination (Everyday Discrimination Scale)	
	Proximal risk factors:	
	- Health-related behaviour: Smoking, alcohol intake, cannabis use, physical activity, weight perception, fruit intake, vegetarian diet, dietary pattern (breakfast, lunch, evening meal), coffee/tea intake, sugary drinks intake	
	- Health care use and related factors: Ability to understand medical information (health literacy), compliance with medication, perceived quality of GP, health care use (GP, specialists, psychological care, alternative health care), health care use in other countries	
	- Working conditions: physical activity at work, work-related recovery opportunities	
**Cardiovascular health**	Proximal risk factors:	Angina pectoris, myocardial infarction, intermittent claudication (Rose Questionnaire), heart failure, cerebrovascular events
	History of high blood pressure/hypercholesterolaemia/diabetes, family history of high blood pressure/hypercholesterolaemia/diabetes/cardiovascular disease/sudden death, fainting history, age of menarche, age of menopause	
**Mental health**	Proximal risk factors:	Depressive disorders (PHQ-9), nicotine use-related disorder (Fagerström), alcohol use-related disorder (AUDIT), cannabis use-related disorder (CUDIT)
	perceived social support (DES subscale), childhood trauma, parental psychiatric history, mastery (Pearlin-Schooler Mastery Scale), neuroticism and extraversion (NEO Five-Factor Inventory), stressful life events	
**Infectious diseases**	Proximal risk factors:	History and presence of allergy, asthma, rhinitis, food allergy, urogenital infections
	Family history of allergy/asthma, travel behaviour (visited other countries), use of self-tests, blood transfusions, use of drugs by injection, surgery in other countries, sexual behaviour, use of contraceptives (women), human papilloma virus (HPV) vaccination (women), circumcision (men)	

**Table 3:** Measurements and collection of biological samples during the physical examination

**Measurements**	**Laboratory measurements**
- anthropometry (weight, height, and circumferences of waist, hip, thigh, arm, and calf),	- fasting blood sample: haemoglobin, HbA1c, glucose, triglycerides, total cholesterol, HDL cholesterol, LDL cholesterol, creatinine
- body fat percentage (using bioelectrical impedance),	- morning urine sample: pH, glucose, ketones, leucocytes, nitrite, protein, and erythrocytes (dipstick), microalbumin, creatinine
- hand grip strength,	
- blood pressure (sitting position) and ankle-arm index (in supine position),	
- arterial stiffness using Arteriograph (oscillometrically measured pulse wave velocity, aortic augmentation index, central systolic blood pressure),	
- heart function using Nexfin (non-invasive haemodynamics such as stroke volume, cardiac output, and systemic vascular resistance),	
- electrocardiogram (left ventricular hypertrophy, infarction, etc.),	
- medication use,	
- health literacy test,	
- respiratory symptoms, vaginal hygiene (women)	
- collection of biological samples (fasting blood sample, morning urine sample, faeces sample, nasal and throat swabs, a vaginal swab in women)	

### Blood sample

A trained research assistant or study nurse collects a fasting venous blood sample. All blood samples are manually processed and aliquoted immediately after collection by a trained technician. Samples of EDTA whole blood and heparin plasma are transported directly to the AMC Clinical Chemistry Laboratory for determination of haemoglobin, HbA1c, glucose, lipid profile, and creatinine levels. The remaining samples are transported to the Durrer Center for Cardiogenetic Research at the AMC, where samples are checked, registered, and stored at -80°C. These samples include EDTA whole blood, EDTA plasma, heparin plasma, serum, and platelet-poor citrate plasma. Genomic DNA is obtained from the buffy coat in the EDTA tube.

### Morning urine sample

Participants are asked to bring a morning urine sample to the physical examination. The urine sample is tested with a dipstick (Combur 7, Roche) on the spot, to determine pH, glucose, ketones, leucocytes, nitrite, protein, and erythrocytes. One sample is transported to the AMC Clinical Chemistry Laboratory for direct analyses of microalbumin and creatinine levels. In addition, together with the blood samples, urine samples are transported to the Durrer Center and stored at -80°C.

### Nasal and throat swabs

A trained nurse or research assistant collects a throat swab during the physical examination visit to the study site. This is done using a dry sterile swab at the posterior nasopharynx, and the swab is then placed in a tube containing viral transport medium. A nasal swab is collected using a dry flocked sterile swab that is rubbed against the turbinate to ensure that the swab contains cells as well as mucus. Subsequently, the swab is inserted into the same tube with transport medium that contains the throat swab. The swabs in the transport medium are kept at room temperature until they are transported to the AMC Department of Medical Microbiology on the same day. The tubes containing swabs and transport medium are vortexed to release all material (cells, mucus) from the swabs into the medium. Subsequently, the transport medium is divided into two aliquots and stored at -80°C for future viral culture and molecular detection of viral and bacterial pathogens.

### Vaginal swab

During the study visit, female participants are asked for a vaginal swab. They are provided with standardized instructions, after which they take a self-administered vaginal swab. A COPAN swab is used, and this is immediately placed into the accompanying plastic tube without fluid. The tubes with dry swabs are kept at +4°C at the research location until they are transported (within two weeks) to the Regional Reference Laboratory at GGD Amsterdam; there they are stored at -20°C for future determination of vaginal microbiome composition and detection of viral and bacterial pathogens.

### Faeces sample

At the end of the physical examination visit, participants are given a faeces collection tube and a safety bag (for transport). They are asked to collect a morning stool sample in the pre-labelled tube, put this in the safety bag, and bring it to the research location within six hours after collection. At the research location, the sample is temporarily stored at -20°C until transportation to the AMC, where the samples are checked by a study nurse and stored at -80°C for later analysis of microbiota composition.

### Further expansion

By February of 2013, approximately 5,000 respondents had been included in HELIUS. The main objective of the study’s next stage is to increase the number of respondents to 10,000 by the end of 2013, with approximately 1,650 participants per ethnic group. The objective of the following stage is to further increase the number to a total of 30,000 respondents.

At five-year intervals, all participants will be invited for a follow-up health examination. During follow-up, baseline measurements will be repeated to study the changes in risk factors and the incidence of cardiovascular disease, mental health disorders, and infectious diseases over time. In addition, participants’ data will be linked to registry data on a continuous basis for routinely collected data on health outcomes and health care (e.g. mortality, hospital admission) at the individual level. This use of existing data has several advantages. After providing adequate informed consent, the active cooperation of the participant is not required, thus reducing the burden for the participant. It also enhances completeness and quality of the data. Finally, it enables us to compare clinical data (including self-reported clinical data) with registry data (e.g. in the case of self-reported diagnoses such as diabetes mellitus and depression). Potentially useful databases/registries to be linked to the HELIUS data collection include local GP registries, hospital discharge registries, pharmacy data (prescriptions), health care insurance registries (health care use), and vaccination registries.

### Statistical power

To illustrate the statistical power of the total HELIUS cohort, with 5,000 participants per ethnic group, we present two examples with different designs. First, we consider cross-sectional research with respect to risk factors for depression with a prevalence of 7% in the general population. With 30,000 participants, the cohort will have at least 90% power to find a significant association between a risk factor and the presence of depression if the odds ratio is larger than 1.20 assuming a risk factor that has relative frequency of 20%. With respect to a difference between ethnic groups in the association of a risk factor with depression, the cohort with 5,000 participants per ethnic group will have at least 90% power to show a difference in odds ratios if the ratio of the odds ratios is 1.95 larger assuming a odds ratio of 1.5 in the reference ethnic group.

As a second example of the statistical power, we consider longitudinal research with respect to a risk factor for acute myocardial infarction (MI). The total incidence in the general population is about 2.5 individuals per 1,000 participants per year, thus after a five year follow-up we will have about 375 participants with acute MI. With this number of cases there would be 90% power to find an association between a risk factor with prevalence of 20% and acute MI if the relative risk (RR) is 1.51. To find a true interaction between such risk factors and acute MI, the ratio of the relative risk must be 2.54.

## Discussion

Ethnic minority groups are excluded from most epidemiological studies in high-income countries in Europe. If they *are* included, the number of respondents from ethnic minority groups is generally too small to draw conclusions about specific ethnic groups. Consequently, the current evidence on the epidemiological profile of ethnic minorities is limited. Against this backdrop, the HELIUS study is unique in its goals and study population. We are not aware of other population-based cohort studies in Europe that include a broad range of ethnic groups, with a substantial number of respondents per group and a diversity of disease phenotypes. In their 2006 review of 72 cardiovascular cohort studies, Ranganathan and Bhopal concluded that even at the international level, only 15 of these were able, by design, to compare different ethnic groups. All of these were performed in the United States. Of the 41 studies in Europe, none was able to provide data according to ethnic group [43].

The association between ethnicity and health may differ between situations, countries, and over time, depending on the characteristics of ethnic groups. If, for example, ethnic inequalities in health in a specific setting have been shown to be related to unhealthy behaviour, this does not necessarily apply to other settings or time periods. Also, the characteristics that link ethnicity to behaviour may differ. Therefore, it is crucial to study the mechanisms that link ethnicity to health if the aim is to understand how ethnic inequalities in common diseases arise. When these mechanisms have been identified, the role of these factors in ethnic inequalities in health may potentially be generalized to other settings.

By introducing ethnic heterogeneity, and by obtaining detailed information on the proximal risk factors and causal mechanisms underlying ethnic inequalities in health, the goal of HELIUS is to provide scientific evidence as to how ethnicity has an impact on the major causes of the global burden of disease. This evidence can be used to develop ethnically targeted interventions, and to target health care to the health needs of ethnic minority populations in high-income countries. HELIUS will thus contribute to the evidence base for the prevention of diseases and promotion of population health in general, and the reduction of ethnic inequalities in health in particular.

## Competing interests

The authors declare that they have no conflict of interest.

## Authors’ contributions

All authors contributed to the conceptual framework, and the development of the design of the HELIUS study. They are all members of the executive board of the HELIUS study. KS drafted the manuscript. MBS, RJP, MP, AHS and AHZ commented on draft versions of the paper. All authors read and approved the final manuscript.

## Pre-publication history

The pre-publication history for this paper can be accessed here:

http://www.biomedcentral.com/1471-2458/13/402/prepub
